# A comparison of the International Consensus and 5th World Health Organization classifications of mature B-cell lymphomas

**DOI:** 10.1038/s41375-022-01764-1

**Published:** 2022-12-02

**Authors:** Brunangelo Falini, Giovanni Martino, Stefano Lazzi

**Affiliations:** 1 Institute of Hematology and CREO, University of Perugia, Perugia, Italy; 2 Institute of Pathology, Department of Medical Biotechnology, University of Siena, Siena, Italy

**Keywords:** Leukaemia, Diagnosis, Haematopoietic system

## Abstract

Several editions of the World Health Organization (WHO) classifications of lympho-hemopoietic neoplasms in 2001, 2008 and 2017 served as the international standard for diagnosis. Since the 4th WHO edition, here referred as WHO-HAEM4, significant clinico-pathological, immunophenotypic and molecular advances have been made in the field of lymphomas, contributing to refining diagnostic criteria of several diseases, to upgrade entities previously defined as provisional and to identify new entities. This process has resulted in two recent classifying proposals of lymphoid neoplasms, the International Consensus Classification (ICC) and the 5th edition of the WHO classification (WHO-HAEM5). In this paper, we review and compare the two classifications in terms of diagnostic criteria and entity definition, with focus on mature B-cell neoplasms. The main aim is to provide a tool to facilitate the work of pathologists, hematologists and researchers involved in the diagnosis and treatment of lymphomas.

## Introduction

Several editions of the World Health Organization (WHO) classifications of lympho-hemopoietic neoplasms in 2001, 2008 and 2016 served as the international standard for diagnosis. However, despite significant progress in the field, there has been no update since the WHO 2016 classification, here referred as WHO-HAEM4. Two classifying proposals of lymphoid neoplasms recently appeared which are hereby named as International Consensus Classification (ICC) and 5th edition of the WHO classification (WHO-HAEM5). The ICC has been published in its definitive format, whilst there only a preview of the WHO Blue Book (which may still be subject to some changes) has appeared. Both ICC and WHO-HAEM5 are based on the same concepts which had inspired the REAL classification [1], e.g., definition of a disease entity based on distinctive pathological, clinical and, when available, molecular features. As compared to WHO-HAEM4 [2], there have been several changes in ICC [3] and WHO-HAEM5 [4] that differ in some structural modifications, terminology and additional/upgrade of new entities defined by clinical, immunophenotypic and molecular data. Unlike ICC, provisional entities are not considered in WHO-HAEM5, whilst some categories regarded as provisional in WHO-HAEM4 have been upgraded to definite entities in both the new classifications. Based on progress in genomic studies, multiple myeloma (MM)/plasma cell myeloma (PCM) has undergone major revisions, to include new distinct cytogenetic entities as reported in ICC. The WHO-HAEM5 also includes a section on “transformations of indolent B-cell lymphoma”, not considered in ICC. In general, diagnostic criteria and recommended ancillary studies have been refined in both classifications. In particular “essential” and “desirable” diagnostic criteria for each entity are discussed in WHO-HAEM5 classification. An important practical issue is how the diagnosis should be reported in everyday work. A reasonable compromise for the time being may be that diagnosis is indicated both according to WHO-HAEM5 and ICC.

For all the above reasons, we here review and compare the two classifications in terms of diagnostic criteria and entity definition, with focus on mature B-cell neoplasms. The main aim is to serve as a tool for pathologists, hematologists and researchers involved in the diagnosis and treatment of lymphomas.

## Mature B-cell neoplasms

### Chronic lymphocytic leukemia/small lymphocytic lymphoma (CLL/SLL)

Diagnostic criteria for CLL/SLL are the same in ICC and WHO-HAEM5 and are based on detection of essential antigens, such as CD19, CD20, CD5, and CD23. Other useful markers include CD43, CD79b, CD81, CD200 and ROR1 [5]. Before starting therapy, the mutational status of *IGHV* and *TP53*/17p alterations should also be assessed [6, 7]. Mutations of *NOTCH1*, *SF3B1* and *BIRC3* [8] may have a prognostic value [9, 10] but their search remains optional [11]. Complex karyotype and *TP53*/17p alterations identify high-risk patients [12] who can benefit from targeted therapies [13]. In the WHO-HAEM5, the term “prolymphocytic progression of CLL” has been introduced. Accelerated CLL [14] should be distinguished from diffuse large B-cell transformation (Richter syndrome), that is characterized by sheets of large cells rather than expanded proliferation centers. Richter-like transformation, incidentally observed after ibrutinib interruption [15, 16] may reflect the emergence of a B-cell clone following the sudden release of B-cell receptor signaling inhibition at drug interruption [16]. This proliferation is abrogated by ibrutinib re-introduction [16].

### B-cell prolymphocytic leukemia (B-PLL)

**Fig. 1:**
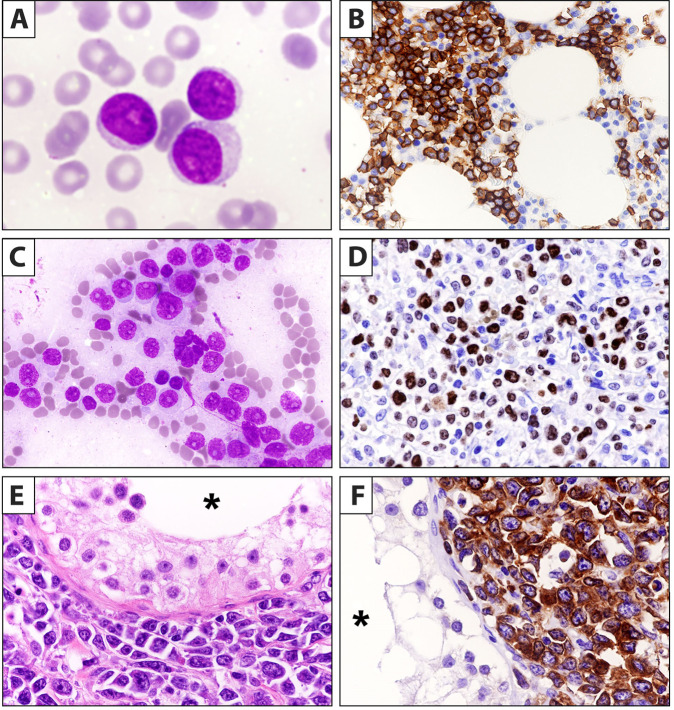
Morphological and immunohistochemical features of B-PLL, pleomorphic variant of classic mantle cell lymphoma and DLBCL of the testis. **A** Peripheral blood smear showing typical prolymphocytes (May-Grunwald-Giemsa; ×1000). **B** Bone marrow trephine showing interstitial infiltration by CD79b positive prolymphocytes (immunoperoxidase staining; ×400). **C** Imprint from spleen involved by classic mantle cell lymphoma, pleomorphic variant (May-Grunwald-Giemsa; ×400). The tumor cells are medium to large in size and contain evident nucleoli. **D** Spleen paraffin section from the same case showing a high percentage of Ki-67 positive cells (immunoperoxidase staining; ×400). **E** Diffuse large B-cell lymphoma of the testis. The asterisk indicates the lumen of a seminiferous tubule (Hematoxylin-eosin; ×400). **F** The same case as **D**, showing strong positivity of tumor cells for CD20 (immunoperoxidase staining; 400); the asterisk indicates the lumen of a seminiferous tubule.

**Fig. 2:**
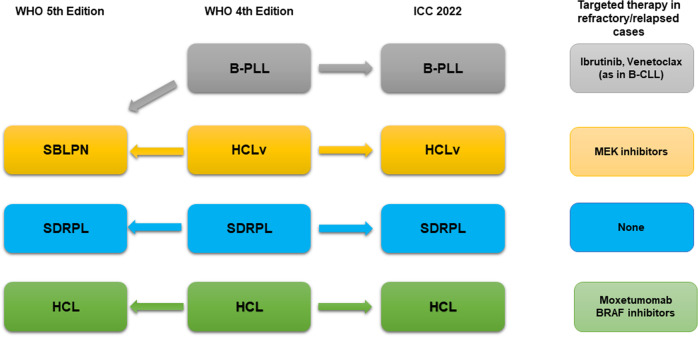
Relationship between different types of B-cell lymphomas with prevalent spleen involvement among the ICC and WHO classifications. B-cell prolymphocytic leukemia (B-PLL), a definite entity in ICC, and hairy cell leukemia variant (HCLv), a provisional entity in ICC, are named in the WHO-HAEM5 under the term of splenic B-cell lymphoma with prominent nucleoli (SBLPN). SBLPN is an heterogeneous category that also comprises cases of unrecognized leukemic mantle cell lymphoma and progressed B-CLL. SDRPL splenic diffuse red pulp small B-cell lymphoma, HCL hairy cell leukemia, SMZL splenic marginal zone lymphoma.

“B-cell prolymphocytic leukemia (B-PLL)” usually occurs in older patients and is characterized by high leukocyte count with >55% prolymphocytes (Fig. 1A), splenomegaly, minimal/absent lymphadenopathy and aggressive course. In the WHO-HAEM5, B-PLL has been deleted as an entity being regarded as a heterogeneous category including cases of hairy cell leukemia variant (HCLv), leukemic mantle cell lymphoma (MCL) and CLL/SLL progressed to B-PLL. Thus, now it has been in part absorbed in the new entity named “splenic B-cell leukemia with prominent nucleoli” (SBLPN) that also includes HCLv (Table 1 and Fig. 2). Conversely, the ICC still regards B-PLL as an entity but recommends its diagnosis only in cases without previous history of B-CLL (to exclude CLL progressing to B-PLL), negative for cyclin D1 and SOX11 (to exclude MCL), and lacking hairy surface projections and intrasinusoidal bone marrow (BM) infiltration (to exclude HCLv and splenic marginal zone lymphoma (SMZL)) (Fig. 1B). B-PLL usually carries a complex karyotype with rearrangement and/or increased copy number of *MYC* (62%), del17p (38%) and trisomy 18 (30%) [17]. B-PLL patients are treated according to B-CLL guidelines. B-PLL harboring *TP53* mutations and/or deletions that predict poor survival usually benefit from bruton tyrosine kinase inhibitors (BTKi) [18, 19]. Patients failing BTKi may still respond to the BCL2 inhibitor venetoclax [20].

**Table 1:** Comparison of the WHO and ICC 2022 classifications of mature B-cell neoplasms.

WHO Classification, revised 4th edition	WHO Classification, 5th edition	ICC 2022
Pre-neoplastic and neoplastic small lymphocytic proliferations		
Monoclonal B-cell lymphocytosis	Monoclonal B-cell lymphocytosis	Monoclonal B-cell lymphocytosis
CLL-type (low and high count)	Low-count or clonal B-cell expansion	CLL-type (low and high count)
	CLL/SLL-type	
- Non-CLL-type	Non-CLL/SLL-type	- Non-CLL-type
- Atypical CLL-type		- Atypical CLL-type
Chronic lymphocytic leukemia /small lymphocytic lymphoma	Chronic lymphocytic leukemia/small lymphocytic lymphoma	Chronic lymphocytic leukemia /small lymphocytic lymphoma
B-cell prolymphocytic leukemia	Entity deleted (renamed Splenic B-cell lymphoma/leukemia with prominent nucleoli)	B-cell prolymphocytic leukemia
Splenic B-cell lymphoma and leukemias		
Splenic marginal zone lymphoma	Splenic marginal zone lymphoma	Splenic marginal zone lymphoma
Hairy cell leukemia	Hairy cell leukemia	Hairy cell leukemia
Splenic B-cell lymphoma/leukemia, unclassifiable	Splenic diffuse red pulp small B-cell lymphoma	Splenic B-cell lymphoma/leukemia, unclassifiable
- Splenic diffuse red pulp small B-cell lymphoma (provisional)	Splenic B-cell lymphoma/leukemia with prominent nucleoli (also includes hairy cell leukemia-variant and cases of B-cell prolymphocytic leukemia)	- Splenic diffuse red pulp small B-cell lymphoma (provisional)
- Hairy cell leukemia-variant (provisional)		- Hairy cell leukemia-variant (provisional)
Lymphoplasmacytic lymphoma and IgM MGUS		
Lymphoplasmacytic lymphoma	Lymphoplasmacytic lymphoma	Lymphoplasmacytic lymphoma
	- IgM-LPL/Waldenstrom macroglobulinemia	
	- non-IgM-LPL/Waldenstrom macroglobulinemia	
IgM MGUS	IgM MGUS (see plasma cell neoplasms)	IgM MGUS
		- Plasma cell type
		- Not otherwise specified (NOS)
Not considered as an entity	Cold agglutinin disease (new entity, not included in this category; see plasma cell neoplasms)	Cold agglutinin disease (new entity)
Marginal zone lymphoma		
Extranodal marginal zone lymphoma of mucosa-associated lymphoid tissue (MALT lymphoma)	Extranodal marginal zone lymphoma of mucosa-associated lymphoid tissue (MALT lymphoma)	Extranodal marginal zone lymphoma of mucosa-associated lymphoid tissue (MALT lymphoma)
Not considered as an entity	Primary cutaneous marginal zone lymphoma (new entity)	Primary cutaneous marginal zone lymphoproliferative disorder (distinct entity)
Nodal marginal zone lymphoma	Nodal marginal zone lymphoma	Nodal marginal zone lymphoma
Pediatric nodal marginal zone lymphoma (provisional)	Pediatric marginal zone lymphoma (distinct entity)	Pediatric nodal marginal zone lymphoma (provisional)
Follicular lymphoma		
Follicular lymphoma	Follicular lymphoma	Follicular lymphoma
- In situ follicular neoplasia	- In situ follicular B-cell neoplasm	- In situ follicular neoplasia
- Duodenal-type follicular lymphoma	- Duodenal-type follicular lymphoma	- Duodenal-type follicular lymphoma
Diffuse follicular lymphoma variant (not considered an entity)	FL with predominantly diffuse pattern (not considered an entity)	BCL2-R-negative, CD23-positive follicle center lymphoma (provisional entity)
Primary cutaneous follicle center lymphoma	Primary cutaneous follicle center lymphoma	Primary cutaneous follicle center lymphoma
Pediatric-type follicular lymphoma	Pediatric-type follicular lymphoma	Pediatric-type follicular lymphoma
Testicular follicular lymphoma	Not considered an entity	Testicular follicular lymphoma (distinct entity)
Mantle cell lymphoma		
In situ mantle cell neoplasia	In situ mantle cell neoplasm	In situ mantle cell neoplasia
Mantle cell lymphoma	Mantle cell lymphoma	Mantle cell lymphoma
Leukemic non-nodal mantle cell lymphoma	Leukemic non-nodal mantle cell lymphoma	Leukemic non-nodal mantle cell lymphoma
Transformations of indolent B-cell lymphomas		
Not included as an entity	Transformations of indolent B-cell lymphomas	Not included as an entity
Large B-cell lymphomas		
Diffuse large B-cell lymphoma, NOS	Diffuse large B-cell lymphoma, NOS	Diffuse Large B-cell lymphoma, NOS
- Germinal Center B-cell subtype	- Recommended	- Germinal Center B-cell subtype
- Activated B-cell subtype	- Recommended	- Activated B-cell subtype
Burkitt-like lymphoma with 11q aberration (provisional entity)	High grade B-cell lymphoma with 11q aberrations	Large B-cell lymphoma with 11q aberration (provisional entity)
Large B-cell lymphoma with IRF4 rearrangement (provisional entity)	Large B-cell lymphoma with *IRF4* rearrangement (upgraded to distinct entity)	Large B-cell lymphoma with *IRF4* rearrangement (upgraded to a distinct entity)
Nodular lymphocyte predominant Hodgkin lymphoma (not included in this category; see Hodgkin lymphoma)	Nodular lymphocyte predominant Hodgkin lymphoma (not included in this category; see Hodgkin lymphoma)	Nodular lymphocyte predominant B-cell lymphoma
T cell/histiocyte-rich large B-cell lymphoma	T-cell/histiocyte-rich large B-cell lymphoma	T cell/histiocyte-rich large B-cell lymphoma
	Primary large B-cell lymphoma of immune-privileged sites	
- Primary diffuse large B-cell lymphoma of CNS	- Primary large B-cell lymphoma of CNS	- Primary diffuse large B-cell lymphoma of CNS
- Not considered as an entity	- Primary large B-cell lymphoma of testis (new entity)	- Primary diffuse large B-cell lymphoma of testis (new entity)
- Reported in primary diffuse large B-cell lymphoma of CNS	- Primary large B-cell lymphoma of vitreoretina	- Included in primary diffuse large B-cell lymphoma of CNS
Primary cutaneous diffuse large B-cell lymphoma, leg type	Primary cutaneous diffuse large B-cell lymphoma, leg type	Primary cutaneous diffuse large B-cell lymphoma, leg type
Intravascular large B-cell lymphoma	Intravascular large B-cell lymphoma	Intravascular large B-cell lymphoma
Not included as an entity	Fluid overload-associated large B-cell lymphoma (new entity)	HHV8 and EBV-negative primary effusion-based lymphoma (provisional entity)
Epstein-Barr virus-positive mucocutaneous ulcer (provisional entity)	Epstein-Barr virus-positive mucocutaneous ulcer (not included in this category; see lymphoid proliferations and lymphomas associated with immune deficiency and dysregulation)	Epstein-Barr virus-positive mucocutaneous ulcer (upgraded to distinct entity)
EBV-positive diffuse large B-cell lymphoma, NOS	EBV-positive diffuse large B-cell lymphoma	EBV-positive diffuse large B-cell lymphoma, NOS
Diffuse large B-cell lymphoma associated with chronic inflammation	Diffuse large B-cell lymphoma associated with chronic inflammation	Diffuse large B-cell lymphoma associated with chronic inflammation
Fibrin-associated large B-cell lymphoma (subtype of DLBCL associated with chronic inflammation)	Fibrin-associated large B-cell lymphoma (new entity)	Fibrin-associated large B-cell lymphoma (subtype of DLBCL associated with chronic inflammation)
Lymphomatoid granulomatosis	Lymphomatoid granulomatosis	Lymphomatoid granulomatosis
Not included as an entity	Described in Lymphoid proliferations/lymphomas associated with immune deficiency and dysregulation (not considered as an entity)	EBV positive polymohrphic B cell lymphoproliferative disorder, NOS (provisional entity)
ALK-positive large B-cell lymphoma	ALK-positive large B-cell lymphoma	ALK-positive large B-cell lymphoma
Plasmablastic lymphoma	Plasmablastic lymphoma	Plasmablastic lymphoma
High grade B-cell lymphoma, with *MYC* and *BCL2* and/or *BCL6* rearrangements	Diffuse large B-cell lymphoma/High grade B-cell lymphoma with *MYC* and *BCL2* rearrangements	High grade B-cell lymphoma with *MYC* and *BCL2* rearrangements
Not included as an entity	Not included as an entity	High grade B-cell lymphoma with MYC and BCL6 rearrangements (provisional entity)
High-grade B-cell lymphoma, NOS	High-grade B-cell lymphoma, NOS	High-grade B-cell lymphoma, NOS
Primary mediastinal B-cell lymphoma	Primary mediastinal B-cell lymphoma	Primary mediastinal B-cell lymphoma
B-cell lymphoma, unclassifiable, with features intermediate between DLBCL and Classic Hodgkin lymphoma	Mediastinal gray zone lymphoma	Mediastinal gray zone lymphoma
KSHV/HHV8-associated B-cell lymphoid proliferations and lymphomas		
Multicentric Castleman disease	KSHV/HHV8 Multicentric Castleman disease (Not included in this category; see lymphoid proliferations and lymphomas associated with immune deficiency and dysregulation)	Multicentric Castleman disease
HHV8-positive germinotropic lymphoproliferative disorder	KSHV/HHV8-positive germinotropic lymphoproliferative disorder	HHV8-positive germinotropic lymphoproliferative disorder
HHV8-positive diffuse large B-cell lymphoma, NOS	KSHV/HHV8-positive diffuse large B-cell lymphoma	HHV8-positive diffuse large B-cell lymphoma, NOS
Primary effusion lymphoma	Primary effusion lymphoma	Primary effusion lymphoma
Burkitt lymphoma		
Burkitt lymphoma	Burkitt lymphoma (emphasis is given in distinguishing EBV+ and EBV- cases)	Burkitt lymphoma
Hodgkin lymphoma		
Classic Hodgkin lymphoma	Classic Hodgkin lymphoma (subtypes maintained as in 4th WHO edition)	Classic Hodgkin lymphoma (subtypes maintained as in 4th WHO edition)
Nodular lymphocyte predominant Hodgkin lymphoma	Nodular lymphocyte predominant Hodgkin lymphoma	Nodular lymphocyte predominant B-cell lymphoma (not included in this category; see large B-cell lymphomas)
Lymphoid proliferations and lymphomas associated with immune deficiency and dysregulation		
Post-transplant	Post-transplant, HIV, Iatrogenic/autoimmune, inborn errors of immunity	Post-transplant
Non-destructive forms distincted in:	Hyperplasia arising in immune deficiency/dysregulation distincted in:	Non-destructive forms distincted in:
- Plasmacytic hyperplasia	- Plasma-cell hyperplasia	- Plasmacytic hyperplasia
- Infectious mononucleosis	- Mononucleosis-like hyperplasia	- Infectious mononucleosis
- Florid follicular hyperplasia	- Follicular hyperplasia	- Florid follicular hyperplasia
Multicentric Castleman disease (not included in this category; see HHV8-associated-lymphoproliferative disorders)	KSHV/HHV8 Multicentric Castleman disease (also included in tumor-like lesion with B cell predominance)	Multicentric Castleman disease (not included in this category; see HHV8-associated-lymphoproliferative disorders)
Polymorphic	Polymorphic LPD arising in immune deficiency/dysregulation	Polymorphic
Epstein-Barr virus-positive mucocutaneous ulcer (not included in this category; see large B-cell lymphoma)	Epstein-Barr virus-positive mucocutaneous ulcer	Epstein-Barr virus-positive mucocutaneous ulcer (not included in this category; see large B-cell lymphoma)
Monomorphic B and T cell neoplasms, cHL	Lymphomas arising in immune deficiency/dysregulation	Monomorphic B and T cell neoplasms, cHL
Lymphomas associated with HIV infection		
Other iatrogenic immunodeficiency-associated LPDs		Other iatrogenic immunodeficiency-associated LPDs
Lymphoproliferative disease associated with primary immune disorders	In born error of immunity-associated lymphoid proliferations and lymphomas	
Plasma cell neoplasms and other diseases with paraproteins		
	Monoclonal gammopathies	
IgM MGUS (Not included in this category; see lymphoplasmacytic lymphoma and IgM MGUS)	IgM MGUS	IgM MGUS (Not included in this category; see lymphoplasmacytic lymphoma and IgM MGUS)
Non-IgM MGUS	Non-IgM MGUS	Non-IgM MGUS
Not considered as an entity	Cold agglutinin disease (see lymphoplasmacytic lymphoma)	Cold agglutinin disease (Not included in this category; see lymphoplasmacytic lymphoma)
Not considered as an entity	Monoclonal gammopathy of renal significant	Not considered as an entity
Heavy chain disease	Heavy chain disease	Heavy chain disease
- Mu heavy chain disease	- Mu heavy chain disease	- Mu heavy chain disease
- Gamma heavy chain disease	- Gamma heavy chain disease	- Gamma heavy chain disease
- Alpha heavy chain disease	- Alpha heavy chain disease	- Alpha heavy chain disease
Plasma cell myeloma	Plasma cell myeloma/Multiple myeloma	Multiple myeloma (plasma cell myeloma)
- Not considered as an entity	- Not considered as an entity	- Multiple myeloma, NOS
- Not considered as an entity	- Not considered as an entity	- Multiple Myeloma with recurrent cytogenetic abnormality
- Not considered as an entity	- Not considered as an entity	- Multiple myeloma with CCND family translocation
- Not considered as an entity	- Not considered as an entity	- Multiple myeloma with MAF family translocation
- Not considered as an entity	- Not considered as an entity	- Multiple myeloma with NSD2 translocation
- Not considered as an entity	- Not considered as an entity	- Multiple myeloma with hyperdiploidy
Solitary plasmacytoma of bone	Plasmacytoma (solitary plasmacytoma of bone, extraosseous plasmacytoma)	Solitary plasmacytoma of bone
Extraosseous plasmacytoma		Extraosseous plasmacytoma
Monoclonal immunoglobulin deposition disease	Disease with monoclonal immunoglobulin deposition	Monoclonal immunoglobulin deposition disease
- Primary amyloidosis	- Immunoglobulin-related (AL) amyloidosis	- Ig light chain amyloidosis (AL)
- Not considered	- Not considered	- Localized AL amyloidosis
- Light chain and heavy chain deposition disease	- Monoclonal immunoglobulin deposition disease	- Light chain and heavy chain deposition disease
Plasma cell neoplasms with associated paraneoplastic syndrome	Plasma cell neoplasm with associated paraneoplastic syndrome	Plasma cell neoplasms with associated paraneoplastic syndrome
- POEMS syndrome	- POEMS syndrome	- POEMS syndrome
- TEMPI syndrome (provisional entity)	- TEMPI syndrome (upgraded to distinct entity)	- TEMPI syndrome
- Not considered as an entity	- AESOP syndrome (new entity)	- Not considered as an entity

### Splenic marginal zone lymphoma (SMZL)

There has been no change in the definition of SMZL in ICC and WHO-HAEM4. SMZL is comprised in the splenic lymphomas/leukemias umbrella of WHO-HAEM5. The diagnosis of SMZL is based on the demonstration of splenomegaly and a BM and/or peripheral blood clonal B-cell population with marginal zone phenotype [21]. The tumor cells carry mutations of *KLF2* [22, 23], *NOTCH2* [23, 24], *TNFAIP3, KMT2D*, and *TP53*, although none of these alterations is specific for SMZL. Search for *MYD88* mutations and immunostaining of BM for the myeloid differentiation nuclear antigen [25] may help to distinguish SMZL from lymphoplasmacytic lymphoma (LPL). Sometimes, the splenectomy specimen is mandatory for the histological differential diagnosis.

### HCL variant (HCLv)

HCLv is a provisional entity which in WHO-HAEM4 and ICC is grouped within the category of “unclassifiable splenic B-cell lymphomas” together with splenic diffuse red pulp small B-cell lymphoma (SDRPL). HCLv is characterized by marked splenomegaly, lymphocytosis, circulating tumor cells with morphology intermediate between hairy cells and prolymphocytes and lack of monocytopenia. Neoplastic B cells express CD11c and CD103 but not CD25 and annexin A1 [26]. *MAP2K1* mutation (p.C121S) has been detected in 7–50% of cases [26, 27] and *BRAF* V600E is usually absent [26, 28]. In the WHO-HAEM5 [4], HCLv is classified as SBLPN because it is biologically different from classic HCL (HCL) (Table 1 and Fig. 2). In the ICC, HCLv remains a provisional entity. Independently by the terminology (HCLv vs. SBLPN) this disease behaves more aggressively than cHL [29] and is resistant to cladribine alone but sensitive to cladribine or bendamustine plus rituximab. The anti-CD22 immunotoxin [30] is potentially effective [29]. *MAP2K1* mutated cases may respond to MEK inhibitors, such as cometinib or trametinib [31].

### Splenic diffuse red pulp small B-cell lymphoma (SDRPL)

SDRPL, regarded as an entity both in WHO-HAEM5 and ICC (provisional), is difficult to diagnose because it shares clinical, morphological and immunophenotypic features with other splenic B-cell lymphomas, especially HCLv/SBLPN. Expression of CD200 [32] and cyclin D3 [33] may help in the differential diagnosis. High frequency of *BCOR* mutations has been detected in SDRPL [34]. However, without splenic tissue demonstrating diffuse infiltration of the red pulp by tumor cells, its distinction from other HCL-like disorders can be very difficult.

### Lymphoplasmacytic lymphoma (LPL) and IgM MGUS

Criteria for diagnosis of LPL have been refined. In the WHO-HAEM4, LPL could be diagnosed when clonal lymphoplasmacytic aggregates represented ≥10% of BM cellularity in trephine biopsies. This percentage is confirmed in the WHO-HAEM5 which recognizes two subtypes of LPL: (1) the IgM-LPL/Waldenström Macroglobulinemia (WM) (about 95%); and (2) the non-WM type LPL (about 5%), including cases with IgG or IgA monoclonal proteins, non-secretory LPL, and IgM-LPL without BM involvement. In ICC, LPL can be diagnosed even when clonal lymphoplasmacytic aggregates represent <10% of BM cellularity in trephine biopsies. In general, confident diagnosis of LPL requires recognition of clonal B cells by flow cytometry and clonal plasma cells by immunohistochemistry, and demonstration of abnormal lymphoplasmacytic aggregates by BM trephine biopsy. *MYD88* (L265P) is the driver mutation of LPL occurring in about 90% of cases [35] and its search is recommended in both classifications, especially to distinguish LPL from nodal and extranodal MZL [36, 37]. However, the absence of *MYD88* mutation does not exclude LPL because a small percentage of cases harbor mutations downstream of MYD88 in the NF-kB signaling pathway [38, 39]. *CXCR4* mutations [40] are found in about 40% of cases and associate with symptomatic hyperviscosity and resistance to ibrutinib [41].

In the WHO-HAEM4, HAEM5 and ICC the diagnosis of IgM monoclonal gammopathy of undetermined significance (MGUS) is made in cases showing <10% of BM neoplastic cells with lymphoplasmacytoid or plasma cell differentiation without lymphoplasmacytic B-cell aggregates diagnostic of LPL [42]. Unlike WHO-HAEM5, the ICC recognizes two IgM MGUS entities [3]: (1) the IgM MGUS of plasma cell type; and (2) the IgM MGUS, not otherwise specified (NOS) (Table 1). The first, represents a precursor of IgM MM and it is characterized by the proliferation of clonal plasma cells without B cells and by the absence of *MYD88* mutation. The t(11;14) *IGH*::*CCND1* or other myeloma-associated *IGH* rearrangements may be present. Conversely, IgM MGUS, NOS is characterized by a proliferation of monoclonal B cells (without lymphoplasmacytic aggregates diagnostic of LPL) usually harboring the *MYD88* mutation. IgM MGUS, NOS may transform into LPL.

Both the ICC and WHO-HAEM5 now recognize primary cold agglutinin disease (CAD) as an entity distinct from LPL or IgM MGUS (Table 1). CAD is a very rare disorder, more frequent in colder countries [43], which is characterized by the proliferation of clonal B cells producing monoclonal cold agglutinins that mediate an autoimmune hemolytic anemia. CAD typically lacks the *MYD88* mutation but displays trisomies of chromosomes 3, 12 and 18 [44], as well as *KMT2D* and *CARD11* mutations [45].

### Extranodal marginal zone lymphoma (MZL) of MALT and nodal MZL

Diagnostic criteria for extranodal and nodal MZL are unchanged. Genetic characteristics of extranodal MZL greatly differ depending on the anatomical site [46]. Therapy also varies among anatomical sites, e.g., gastric MALT may benefit from anti-microbial treatment. Despite these differences, neither ICC nor WHO-HAEM5 classify extranodal MZL based on the site of presentation, the only exception being cutaneous MZL (see below). Nodal MZL is a clinically and molecularly heterogeneous entity. Immunostaining for IRTA1 [47] may help in the diagnosis [48, 49]. Recognition of large cell transformation of MZL is based on the detection of aggregates of large B cells.

Pediatric nodal marginal zone lymphoma (PNMZL) considered in the WHO-HAEM4 and in ICC [3] as provisional entity is upgraded in WHO-HAEM5 [4] to a distinct entity. It usually presents with localized disease and can be rarely observed even in adults. Surgical excision is curative in most patients [50]. Distinction of PNMZL from reactive conditions may require demonstration of clonal *IG* gene rearrangements. Cases with overlapping features between PNMZL and pediatric-type follicular lymphoma have been reported [50].

Both the WHO-HAEM5 [4] and ICC [3] recognize “primary cutaneous marginal zone lymphoma (PCMZL)” as a distinct entity (Table 1). PCMZL resembles morphologically and immunophenotypically nodal and extranodal MZL. However, gain of 6p and loss of 6q (as observed in MZL of ocular adnexa) or the presence of *BIRC3-MALT1* fusions (as seen in gastric and pulmonary MZL) are not found. In ICC, PCMZL is called “primary cutaneous marginal zone lymphoproliferative disorder” [3] rather than lymphoma because of its slow growing characteristics, low propensity for systemic dissemination, and good survival with conservative therapy, although cutaneous recurrences are frequent. The WHO-HAEM5 continues to use the term “PCMZL” [4] and to group it among MZL.

Two subtypes of PCMZL/lymphoproliferative disorder are recognized [51]. About 75% of cases express IgG, especially IgG4 (about 50% of cases) [52, 53] without association with pre-existing systemic IgG4-related disease [52]. Conversely, non-cutaneous MZL only rarely express IgG4. This subtype is characterized by the presence of abundant reactive helper type-2 T cells [54], plasma cells located at the periphery of the lymphoid infiltrate and B cells lacking CXCR3 expression. About 25% of cases show a MALT-lymphoma-like IgM+ phenotype, a T-helper type 1-driven process that occurs in older individuals, involves the subcutis, causes follicular colonization and lacks *MYD88* mutation. In this setting, non-cutaneous primary disease should be excluded. The molecule IRTA1 [47, 49] may help in identifying these cases [55]. About 63.2% of PCMZL/lymphoproliferative disorder carry dominant-negative mutations involving the death domain of the apoptosis-regulating FAS/CD95 protein [56]. *FAS* mutations point to an apoptosis defect that may explain the slow growth and indolent course of this tumor [56]. Mutations of *SLAMF1*, *SPEN*, and *NCOR2* genes have also been reported [56].

### Follicular lymphoma (FL)

The WHO-HAEM5 describes three morphological variants of FL: (1) c*lassic FL* characterized by follicular growth of centrocytes and centroblasts and t(14;18)(q32;q21)/*IGH*::*BCL2* fusion; (2) follicular large B-cell lymphoma corresponding to the WHO-HAEM4 FL grade 3B that rarely carries the *BCL2* translocation. The follicular pattern often co-exists with area of DLBCL. When MUM1/IRF4 is strongly expressed, FISH analysis for IRF4 is required to exclude large B-cell lymphoma with *IRF4* rearrangement; and (3) FL with uncommon features comprising FL with “blastoid” or “large centrocyte” features characterized by high proliferative index, and MUM1/IRF4 expression [57] that needs to be distinguished from large B-cell lymphoma with *IRF4* rearrangement [58]. FL with predominantly diffuse growth pattern [59] is considered a subtype of classic FL that frequently occurs in the inguinal region and likely corresponds to the ICC entity named *BCL2*-R-negative, CD23-positive follicle center lymphoma [3] (see below).

In the ICC, the consensus was to retain the morphological grading of FL, as defined in the WHO-HAEM4, although it remains unclear whether clinically the grade 3A differs from grades 1 and 2 [60]. Conversely, patients with grade 3B FL are usually treated as DLBCL [61, 62]. Hence, distinction between grade 3A and 3B is critical [57]. *BCL2*-rearrangement and CD10 positivity both favor FL grade 3A. In WHO-HAEM5, grading of FL, is regarded as optional because of its scarse reproducibility [63] and questionable clinical significance [64]. The WHO-HAEM4 entity named in situ follicular neoplasia remain unchanged in ICC classification, while in the WHO-HAEM5 it is named in situ follicular B-cell neoplasm. Primary cutaneous follicular lymphoma and duodenal follicular lymphoma remain unchanged in the WHO-HAEM5 and ICC classifications. Pediatric-type FL also remains a distinct entity that usually presents as localized disease in the neck lymph nodes, mostly in young males, being characterized by blastoid cytology, high proliferation rate, lack of BCL2 protein and t(14;18) [65]. MAPK pathway mutations are frequent in pediatric-type FL and the prognosis is excellent following conservative management [65–67]. Distinguishing this entity from FL grade 3B remains critical.

Compared to WHO-HAEM4, there are two changes in the ICC but not in the WHO-HAEM5 concerning FL: (1) testicular FL is recognized as a distinct form of FL in young boys; and (2) *BCL2*-R-negative, CD23-positive follicle center lymphoma [3] is recognized as provisional entity (Table 1). Testicular FL shares clinico-pathological features with pediatric-type FL [68, 69]. In particular, it occurs in children, is limited to testis and usually shows a grade 3 [69]. It also lacks BCL2 expression at immunohistochemistry and *BCL2* rearrangements at FISH. Resection, sometimes followed by two cycles of chemotherapy, results in an event-free survival of 100% at a median follow-up of 73 months [69].

Follicle center lymphoma lacking BCL2 rearrangement and expressing CD23 (a surrogate for *STAT6* mutations) [3] can be diffuse and purely follicular (about one-third of cases). Although it often presents in the inguinal region, it may also occur in axillary and cervical regions as limited stage disease with favorable prognosis. As opposed to conventional FL, it often presents in women. Molecularly, it shows absence of *IGH::BCL2* fusion, and carries frequent *STAT6* and *CREBBP* mutations [59] along with 1p36 deletion [70] or *TNFRSF14* mutations [71].

Routine molecular testing is not necessary in FL but advisable under certain circumstances. *MAP2K1* mutations favor a diagnosis of pediatric-type FL [67]. Detection of *EZH2* mutations is important when therapy with an *EZH2* inhibitor is considered [72]. Integration of mutational analysis (m7-FLIPI) in the risk stratification of FL remains investigational [73]. The activity of CAR-T cells [74] against FL is also independent by the underlying genetic landscape.

### Mantle cell lymphoma (MCL)

Both ICC and WHO-HAEM5 subdivide MCL into: (1) in situ MCL neoplasm (WHO-HAEM5)/in situ mantle cell neoplasia (ICC); (2) conventional (cMCL); and (3) leukemic non-nodal MCL (Table 1). In situ mantle cell neoplasm/neoplasia represents an incidental finding characterized by colonization of mantle zone of B-cell follicles by neoplastic B cells carrying the IGH::*CCND1* fusion and overexpressing cyclin D1 at immunohistochemistry.

cMCL is the most common variant. The *IGH*::*CCND1* fusion generated by t(11;14)(q13;q32) is the genetic hallmark of MCL that is detectable in >95% of cases, *IGK* or *IGL* serving as fusion partners in rare cases. Definition of cMCL has been expanded to include cases lacking *CCND1* rearrangements but harboring cryptic rearrangements of *IGK* or *IGL* enhancers with *CCND1* or translocations involving *CCND2* or *CCND3* [75–78]. The latter are better detected by FISH or mRNA overexpression than immunohistochemistry. *CCND1* rearrangement can also be found in DLBCL raising problems in the differential diagnosis with MCL of plemorphic type. These cases should not be classified as cMCL. Conversely, *MYC* may be rearranged in *bona fide c*MCL, usually with blastoid/pleomorphic morphology [79].

Clinically staging of MCL is based on simplified or combination Mantle Cell Lymphoma International Prognostic Index [80, 81]. In the era of new targeted therapies, including BTKi [82, 83] and CD19-directed CAR-T cells [81, 84], every case of MCL should be investigated prior therapy for morphology (blastoid/pleomorphic variant) (Fig. 1C), immunophenotype, proliferative index (labeling of Ki67) (Fig. 1D) and presence of *TP53* deletions/mutations since the occurrence of one or more of these variables associates with a poor prognosis [81, 85]. Notably, *TP53* deleted/mutated cases are usually resistant to chemotherapy but are sensitive to BTKi [82, 83] or CAR-T cells [81]. Over time, neoplastic cells may acquire mutations conferring resistance to BTKi or venetoclax [86]. Searching these mutations is useful to further guide therapy. Genomic studies point to a high complexity of MCL [87] with possible prognostic impact but additional studies are required before this information is incorporated into classifications.

Leukemic non-nodal MCL is an indolent disease characterized by involvement of BM, spleen and peripheral blood by low proliferating tumor cells and an outcome more favorable than that of cMCL [81]. The diagnosis is favored by the finding of no/limited stage nodal disease, no/low SOX11 expression [88–90], high load of somatic *IGHV* hypermutations (>98%) [89, 91] and lack of *ATM* mutations/deletions or *TP53* mutations [87, 88]. Non-nodal MCL can progress to aggressive cMCL with rapidly enlarging lymphadenopathy following acquisition of *TP53* and/or *ATM* mutations/deletions that confer a poor outcome.

### Transformations of indolent B-cell lymphomas

This entity is only considered in WHO-HAEM5 but not in ICC. The term transformation refers to the emergence, following acquisition of additional genetic aberrations, of an aggressive lymphoma in a patient with previously or synchronously diagnosed, clonally-related low-grade B-cell lymphoma [4] (e.g., Richter transformation, transition from FL or MZL to DLBCL). Usually the transformed lymphoma cells retain the immunophenotype of their low-grade counterparts. Transformed lymphomas can manifest as either nodal and/or extranodal disease. The suspicion of transformation can be heralded by enlarging lymph nodes, increasing LDH and/or appearance of systemic symptoms. Observation of the tissue architecture is critical to establish the diagnosis of transformation. Thus, the fine needle aspiration biopsy is not adequate for this purpose.

### Diffuse large B-cell lymphoma (DLBCL)

DLBCL not otherwise specified (NOS) represents a heterogeneous entity in terms of morphological features (e.g., centroblastic, immunoblastic), immunophenotype (e.g., CD5+, double MYC/BCL2 expressor) [92–94] and cytogenetic/molecular categories [95]. When the diffuse growth pattern cannot be recognized (e.g., fluid-overload-associated large B-cell lymphoma), the WHO-HAEM5 prefers the term of “large B-cell lymphoma”. Most DLBCL, NOS recapitulate the differentiation/maturation processes occurring within the germinal centers (GCs). Cases whose cell-of-origin (COO) is from GC (GCB subtype) show unique gene expression profile [96] and express GC markers at immunohistochemistry. They are also enriched for *IGH::BCL2* fusion and mutations of genes involved in GC development, such as *EZH2*, *GNA13*, *MEF2B*, *KMT2D*, *TNFRSF14*, *B2M* and *CREBBP* [95]. DLBCL deriving from B cells that exit from the GC or from post-GC B cells (activated B-cell-like [ABC] subtype), are characterized by dependence on BCR and NFκB signaling, IRF4/MUM1 expression [97] and enrichment for BCR pathway mutations (e.g., *MYD88*, *CD79B*, *PIM1*) and *PRDM1/BLIMP1* mutations/deletions [95].

Both ICC and WHO-HAEM5 recommend the COO of DLBCL should be retained due to its potential prognostic impact [98]. Because of its simplicity, rapidity and low cost, immunohistochemistry is currently the most widely used method in routine practice. However, it cannot recognize the “unclassified” GEP category. In particular, the application of immunohistochemical algorithms [98] to upfront clinical trials of DLBCL, NOS incorporating targeted agents such as bortezomib and ibrutinib have led to disappointing results [99, 100], with the exception of lenalidomide [101]. Thus, COO does not appear to fully capture the high biological complexity of DLBCL. This underlines the importance of moving to a more molecularly-based subdivision of DLBCL. This approach recently led to identifying new genetic subgroups of DLBCL [102–104]. However, the functional significance of the driver mutations defining these clusters and their impact on outcome and targeted therapy remains uncertain. Hopefully, in the future such a molecular information may be combined with COO to improve the risk stratification of DLBCL patients for clinical trials [105]. The activity of anti-CD19 CAR-T cells in relapsed/refractory DLBCL [106] seems to be independent from COO and molecular category.

Primary DLBCL of the central nervous system (PCNSL) and primary DLBCL of testis share many features, including the ABC COO and the high frequency of *MYD88* and *CD79b* mutations [105, 107–109]. Similarly to CNS, the testis is an immune-privileged site where tumor-infiltrating host immune cells play an important role in immune evasion [110, 111]. Moreover, mechanisms defending the developing gametocytes in the immune-privileged site of the testis may provide DLBCL with an ideal milieu for acquiring an immune-escape phenotype [112]. Thus, primary DLBCL of the testis is now considered by ICC as a distinct entity (Fig. 1E, F). Moving from the same concept, WHO-HAEM5 adopted the term of Large B-cell lymphomas of immune-privileged sites to group cases arising in immune sanctuaries defined by the blood-brain, blood-retinal and blood-testicular barriers [107] (Table 1). Interestingly, CD19-directed CAR-T cells were shown to cross the blood-brain barrier [113, 114]. Primary testicular DLBCL is a rare entity characterized by an aggressive course and tendency to relapse in the controlateral testis and CNS [115]. Primary DLBCL, leg and breast type, intravascular large B-cell lymphoma, and primary adrenal lymphomas show features similar to lymphomas of CNS and testis [116–118]. However, ICC and WHO-HAEM5 felt that lumping these tumors under the umbrella term of “extranodal lymphoma ABC type” was premature since they are rather heterogeneous.

**Fig. 3:**
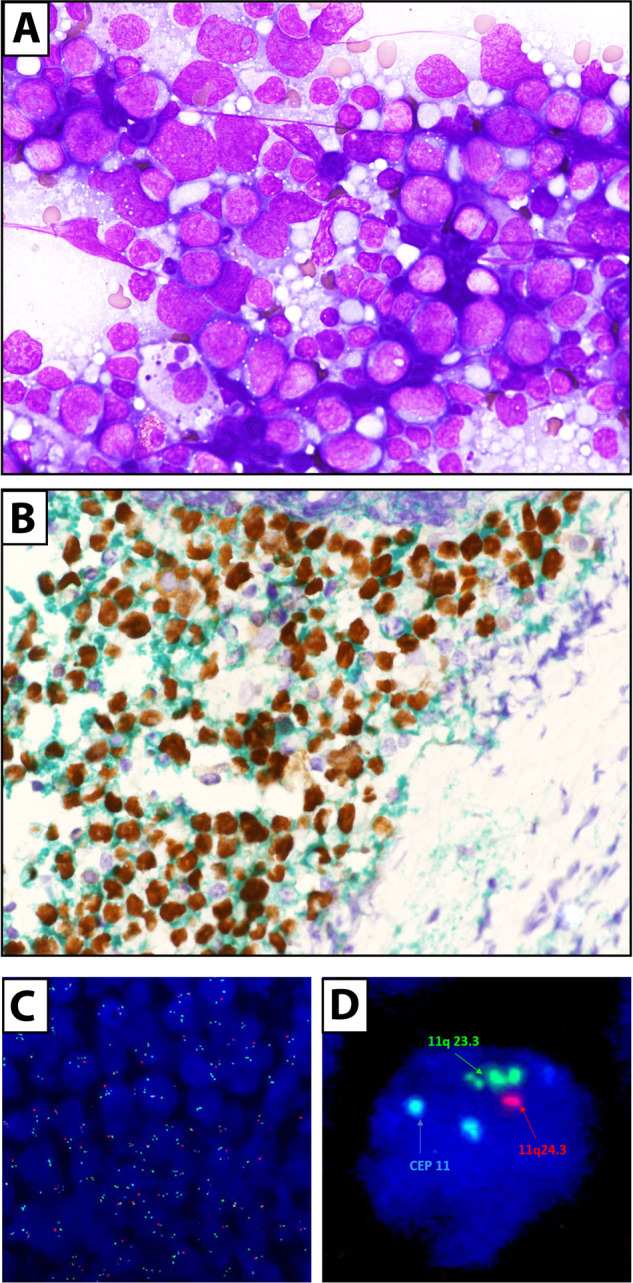
DLBCL/HGBCL with 11q rearrangements. **A** Lymph node imprint showing large-size tumor cells with basophilic cytoplasm and round nuclei with evident nucleoli (May-Grunwald-Giemsa; ×400). **B** Tumor cells are double stained for CD20 (green) and BCL6 (brown). **C**, **D** FISH reveals 11q aberrations.

The WHO-HAEM4 provisional entity “Burkitt-like lymphoma with 11q aberration” was named as such because of its clinical, morphological (“starry sky pattern”) and immunophenotypic (CD10+/BCL6+/BCL2−/Ki67 high) resemblance to Burkitt lymphoma but lack of *MYC* rearrangement [119–121]. These cases are characterized by 11q aberration, often consisting of a gain in 11q23.2–23.3 followed by a telomeric loss in 11q24.1-qter. Subsequently, they were found to carry DLBCL-associated mutations (e.g., *GNA13*) without the hallmark Burkitt lymphoma mutations involving ID3-TCF3 or the SWI/SNF complexes [122, 123]. Due to molecular studies showing that this entity is closer to DLBCL than to Burkitt lymphoma, the ICC recognizes it as provisional entity, under the term of “large B-cell lymphoma with 11q aberration” (Fig. 3). Conversely, because of the frequent intermediate/blastoid morphology with starry sky macrophages [124], the WHO-HAEM5 classifies it as “high-grade B-cell lymphoma with 11q aberration” [125]. Chromosome 11q gains/losses can be detected using FISH but chromosomal microarray should be performed if FISH is equivocal. The best therapeutic option for these cases remains uncertain. In pediatric patients, large/high-grade B-cell lymphoma with 11q aberration is less frequent than *MYC* breakpoint-positive Burkitt lymphoma, occurs at older age, shows less male predominance, lower LDH, less abdominal involvement and it is characterized by an excellent prognosis.

Large B-cell lymphoma with *IRF4* rearrangement was introduced in the WHO-HAEM4 as a provisional entity under follicular lymphoma and is retained there in the ICC. In the WHO-HAEM5 has been moved, most probably because of the name, to the group of aggressive lymphomas, although this presents in children and young adults with localized disease and excellent outcome [126]. The diagnosis is based on demonstration of *IRF4* rearrangement at FISH analysis. *IRF4* mutations can also be detected [126]. This entity does not include aggressive B-cell lymphomas with *IRF4* rearrangements that also harbor *BCL2* and/or *MYC* rearrangements.

### Large B-cell lymphoproliferative disorders related to viral agents according to ICC

The EBV-positive polymorphic B-cell lymphoproliferative disorder, NOS has been added as new entity in ICC. This term should be reserved to cases with/without immunodeficiency  showing an alteration of lymph node architecture due to an EBV-positive polymorphic infiltrate that does not fulfill the criteria for diagnosis of lymphoma [127, 128]. In samples without distortion of the nodal architecture by EBV-positive B cells, the term EBV reactivation is recommended [3]. This entity differs from EBV-positive DLBCL, NOS defined by >80% EBV+ cells that can occur at any age and shows a wide morphological spectrum ranging from monomorphic to polymorphic and an aggressive clinical course [129, 130]. Separating this entity from EBV+ classic Hodgkin lymphoma (cHL) may be problematic. Demonstration of >1 B-cell marker in a significant percentage of tumor cells, extranodal presentation and/or EBV latency III favors the diagnosis of EBV-positive DLBCL, NOS.

EBV-positive mucocutaneous ulcer (EBVMCU) [131–134] has been upgraded from provisional to distinct entity in ICC [131]. The WHO-HAEM5 includes this entity and the EBV-positive polymorphic B-cell lymphoproliferative disorder, NOS in the “Lymphoid proliferations and lymphoma associated with immune deficiency and dysregulation” (Table 1). EBCMCU usually presents in elderly (median age 71 years) as unifocal cutaneous or mucosal ulcer (frequently in the oropharyngeal mucosa probably due to the release of virus into saliva) without associated lymphadenopathy or systemic symptoms [134]. Involvement of the gastrointestinal tract is less frequent and may be preceded by an inflammatory bowel disease [135]. EBVMCU often occurs after a local trauma including tooth extraction and develops in immunocompromised patients, e.g., after therapy with methotrexate [133, 136] or in organ transplant recipients [137]. EBVMCU has a wide morphological spectrum and may simulate DLBCL or even cHL. Focal necrosis and angioinvasion are frequently seen. When >2 skin lesions are present, the term EBV-positive B-cell polymorphic LPD, or if appropriate, EBV-positive DLBCL, NOS should be applied [133]. EBV DNA is usually not detectable in the serum despite the biopsies showing strong EBER-ISH positivity of atypical B cells [137]. EBVMCU is usually characterized by spontaneous regressions or remission after discontinuation or dose reduction of immunosuppressive therapy [132]. However, rituximab and rarely chemotherapy may be required to induce remission [134]. Unlike EBV-positive DLBCL, NOS the prognosis is very good.

### Immunodeficiency-associated lymphoproliferative disorders according to WHO-HAEM5

The WHO-HAEM5 introduces the term immunodeficiency and dysregulation associated lymphoproliferative disorders (IDD-LPSDs) that considers the following aspects for determining an integrated nomenclature [4]: (1) histological diagnosis (hyperplasia, polymorphic LPD, lymphoma as for immunocompetent patients); (2) presence or not of virus (EBV, KSHV/HHV8); (3) clinical setting/immunodeficiency background (post-transplant, HIV, iatrogenic/autoimmune); and (4) inborn errors of immunity. EBV-positive polymorphic B-cell lymphoproliferative disorder, NOS according to ICC and the EBV+ mucocutaneous ulcer are grouped herein. The relationships with other entities of WHO-HAEM 4 and ICC are shown in Table 1.

The new entity defined as “HHV-8- and EBV-negative primary effusion-based lymphoma” provisional in ICC and “Fluid overload-associated large B-cell lymphoma” defined in WHO-HAEM5 occurs in older males without underlying immunodeficiency and presents with exclusive involvement of body cavities, usually pleura [138, 139]. Patients usually have an underlying pathological condition leading to fluid overload, such as chronic heart failure, renal failure, protein-losing enteropathy or liver failure/cirrhosis. Fluid overload is thought to lead to lymphoma through chronic serosal stimulation. Most cases have been reported from Japan and frequently had a history of hepatitis C infection [138]. It often shows a centroblastic rather than plasmablastic morphology, with expression of at least one B-cell antigen and a GCB COO. It exhibits better prognosis than primary effusion lymphoma, with spontaneous regression or cure following drainage alone [140]. Unlike WHO-HAEM5, the ICC does not accept EBV+ lesions since they are usually associated with immunosuppression and therefore belong to a different category. The fibrin-associated large B-cell lymphoma, regarded in the WHO-HAEM4 and ICC as a subtype of DLBCL-associated with chronic inflammation, is now upgraded to a definite entity in the WHO-HAEM5.

### High-grade B-cell lymphomas (HGBCL)

The WHO-HAEM4 included: (1) HGBCL with *MYC* and *BCL2* and/or *BCL6* rearrangements (“double-hit” or “triple-hit”); and (2) HGBCL, NOS. In ICC, “double-hit” HGBCL now comprises two entities: (1) HGBCL with *MYC* and *BCL2* rearrangements (with or without *BCL6* rearrangement) (HGBCL-DH-*BCL2*); and (2) a provisional entity named “HGBCL with MYC and BCL6 rearrangements (HGBCL-DH-*BCL6*)” (Table 1). In WHO-HAEM5, tumors with MYC and BCL2 rearrangements are named diffuse large B-cell lymphoma/high-grade B-cell lymphoma with *MYC* and *BCL2* rearrangements (DLBCL/HGBCL MYC/BCL2).

DLBCL or HGBCL with *MYC* and *BCL2* rearrangements show a morphology ranging from large cells to blastoid/intermediate. FISH break apart probes are recommended for diagnosis but they may miss the rearrangement in up to 20% of cases. This entity is sometimes preceded by a history of FL and shows a gene expression profile close to centroblasts of the GC dark zone [104, 141, 142]. Moreover, it exhibits a mutational signature more similar to FL (C*REBBP, BCL2, KMT2D, MYC, EZH2* and *FOXO1* mutations) than to DLBCL, NOS (GCB subtype) [143]. Thus, it differs biologically from HGBCL-DH-BCL6 [144]. It also frequently carries *MYC* hotspot mutations affecting the phosphorylation site and its adjacent amino acids, which are important for MYC protein degradation, resulting in higher MYC expression that may be responsible for the aggressive clinical behavior, [142, 145]. Cases carrying *MYC* and *BCL2* abnormalities other than typical translocations [146] should not be currently classified as DLBCL- or HGBCL-DH-*BCL2*.

HGBCL-DH-*BCL6* is characterized by frequent involvement of extranodal sites, aggressive clinical course and poor prognosis [144, 147]. As compared to DLBCL or HGBCL with *MYC* and *BCL2* rearrangements, it shows GCB immunophenotype less often, is more likely to be CD10(−)/IRF4/MUM1(+), infrequently expresses BCL2 and is cytogenetically less complex [144]. Moreover, it does not show the impairment of *TP53* and *MYC* signaling pathway typically observed in DLBCL or HGBCL with *MYC* and *BCL2* rearrangements, whereas it exhibits impairment of E2F targets [148]. Because of its less distinctive biological features [141], HGBCL with *MYC* and *BCL6* rearrangements, regarded as a provisional entity in ICC, is considered in the WHO-HAEM5 as a genetic subtypes of DLBCL, NOS and HGBCL, NOS, respectively.

HGBCL, NOS defines a rare molecularly heterogeneous subset of cases with blastoid or Burkitt-like cytology that do not have double-hit cytogenetics and do not readily fit within the categories of DLBCL, NOS or Burkitt lymphoma [149]. HGBCL, NOS usually shows a GCB phenotype and about half of cases carry a single-hit MYC rearrangement. Clinically, it occurs mostly in older adults (median age, 70 years) and it is characterized by high LDH, high IPI, extranodal involvement, CNS invasion and a more aggressive behavior than DLBCL [150]. Optimal treatment of HGBCL, NOS, remains uncertain because of the rarity of the tumor and the diagnostic variability. Patients presenting with aggressive disease should be treated with intensified Burkitt lymphoma-like regimens [150]. HGBCL also appears to respond well to immunotherapy with CAR-T cells [151].

**Fig. 4:**
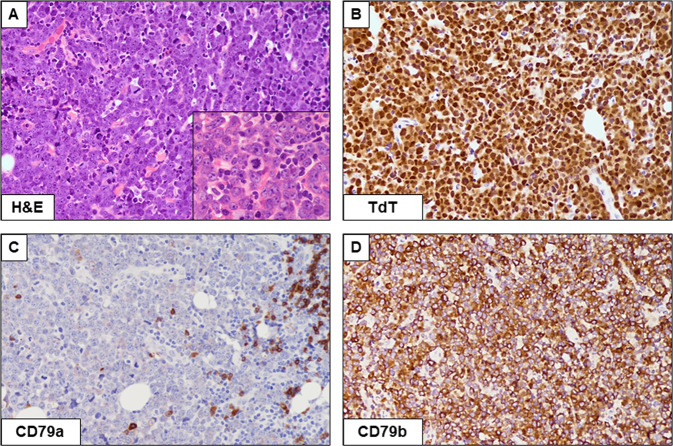
High-grade B-cell lymphoma of the heart with *MYC* and *BCL2* rearrangement. **A** Diffuse infiltration by large-size tumor cells (Hematoxylin-eosin; ×400) with evident nucleoli (inset; ×600). **B** The majority of the neoplastic cells strongly express TdT. **C**, **D** Tumor cells are CD79a negative but strongly express CD79b (TdT, CD79a, CD79b, Immunoperoxidase staining; ×400).

In the WHO-HAEM4, cases with HGBCL (or DLBCL) morphology expressing TdT were classified as lymphoblastic leukemia/lymphoma. Now, based on mutational studies, CD34 negativity and presence of isolated or double-hit MYC rearrangement [152, 153], both WHO-HAEM5 and ICC recommend to consider these cases as DLBCL or HGBCL, NOS with “expression of TdT” (Fig. 4).

### Burkitt lymphoma (BL)

The definition of BL remains unchanged in WHO-HAEM5 and ICC. BL is a very aggressive tumor characterized by a monotonous proliferation of medium-sized tumor cells, often associated with a “starry sky” pattern, GCB phenotype (CD10+, BCL6+, BCL2−), high proliferative index (Ki67 > 90%), *IG*::*MYC* juxtaposition and mutations involving *TCF3 (E2A)* or its repressor *ID3* [154]. Three subtypes of BL are recognized in WHO-HAEM4: (1) “endemic”; (2) “non-endemic or sporadic”; and (3) “immunodeficiency-associated”. WHO-HAEM5 believes BL is better defined by molecular features than by epidemiologic criteria/geographic location [155–158]. Therefore, it emphasizes the distinction of BL in two subtypes: EBV-positive BL and EBV-negative BL, to reflect the dual mechanism of BL pathogenesis, i.e., virus-driven versus mutational-driven. EBV-positive cases exhibit higher levels of somatic hypermutation particularly in non-coding sequences close to the transcription start site [156] and harbor fewer driver mutations, including those affecting *TCF3* and *ID3* [156]. ICC recommends to classifying BL cases expressing TdT as B-lymphoblastic leukemia/lymphomas with *MYC* rearrangement rather than BL. In fact, they display phenotypic and molecular features of precursor B cells, including IG::*MYC* translocations arising from aberrant VDJ recombination, frequent lack of a productive IGH rearrangement and recurrent *NRAS*/*KRAS* mutations [159]. Separating these cases from BL is clinically important.

### Hodgkin lymphoma

cHL is a GC-derived tumor characterized by a low number of neoplastic Hodgkin and Reed-Sternberg (H-RS) cells with a defective B-cell program that are immersed in an immunosuppressive microenvironment [160]. Genomic studies on microdissected tumor cells showed deregulation of the JAK-STAT pathway due to genetic alterations in *STAT3*, *STAT5B*, *JAK1*, *JAK2*, and *PTPN1* in about 90% of cHL [161]. This finding supports the role of JAK-STAT pathway activation [161], among other genetic alterations [160], in cHL pathogenesis. In both ICC and WHO-HAEM5, diagnostic criteria for cHL remain unchanged. Immunohistochemical detection of CD30, CD15, IRF4/MUM1, PAX5, CD20, CD3 and LMP1 or EBER in situ hybridization is recommended. When H-RS cells are numerous and express CD20, cases should be investigated for the presence of additional B-cell markers, to exclude mediastinal gray zone lymphoma (MGZL). Moreover, cHL should be distinguished from other mimickers, e.g., lymphoproliferative disorders arising in the context of immune deficiency, that may contain EBV-positive H-RS-like cells [3, 4].

In ICC, the term nodular lymphocyte predominant Hodgkin lymphoma (NLPHL) has been changed into that of nodular lymphocyte predominant B-cell lymphoma. Conversely, WHO-HAEM5 felt the change of terminology was still premature and preferred to maintain the term of NLPHL, also to avoid interference with ongoing clinical trials [4]. The ICC decision was based on major biological and clinical differences between NLPHL and cHL, e.g., retention of functional B-cell program in NLPHL and close relationship of NLPHL to T-cell/histiocyte-rich large B-cell lymphoma [162, 163]. ICC also advises that “Fan patterns” A, B and C or Grade 1, should be distinguished from the less common “Fan patterns” D, E and F or Grade 2 [164]. The latter are usually associated with loss of nodularity, increased infiltration by T cells and a more aggressive clinical course. Cases with grade 2 histology may warrant treatment as DLBCL and patients with advanced stage NLPHL respond well to CHOP regimen [165]. Rare cases of NLPHL are EBV+ but the clinical significance of this finding remains unclear [166].

### Mediastinal gray zone lymphoma (MGZL)

MGZL shows overlapping features with primary mediastinal B-cell lymphoma (PMBL) and cHL (especially nodular sclerosis). In both ICC and WHO-HAEM5, this entity now replaces the term “B-cell-lymphoma, unclassifiable with features intermediate between DLBCL and classic Hodgkin lymphoma” of WHO-HAEM4. Evidence that MGZL represents a true biological continuum with cHL and PMBL rather than a morphological mimic is supported by immunophenotypic patterns, gene expression profiles, methylation and mutational studies showing intermediate biological features between cHL and PMBL [167–170]. The concept of MGZL is further reinfocsed by the finding that nodular sclerosis cHL and PMBL developing sequentially may have common clonal origin [171]. These findings most likely reflect derivation of cHL, PMBL and MGZL (all located in the anterior mediastinum) from thymic B cells [172].

**Fig. 5:**
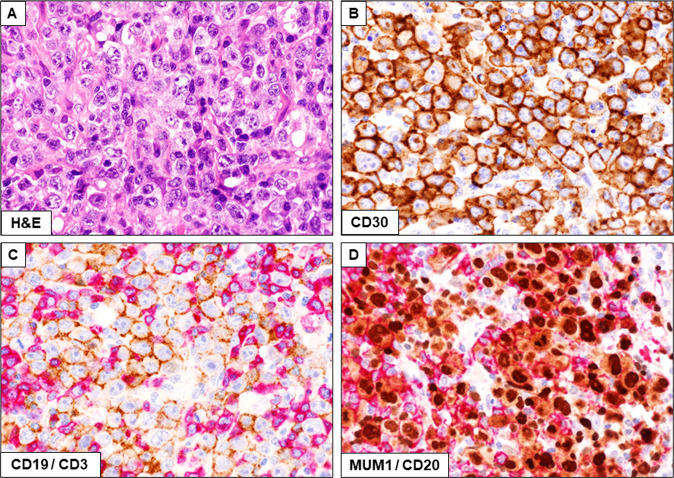
Mediastinal gray zone lymphoma. **A** Diffuse proliferation of large tumor cells with wide clear cytoplasm (Hematoxylin and eosin; ×400); **B** Neoplastic cells strongly express CD30 (immunoperoxidase staining ×400). **C** Double staining showing a small percentage of reactive CD3-positive T cells (red) together with many surface CD19-positive tumor cells (brown); **D** Tumor cells from the same case are also double stained for MUM1/IRF4 (brown) and surface CD20 (red) (×400) (**C**, **D** Double immunoperoxidase staining using different enzyme substrates).

MGZL most frequently presents in young men (average 30 years) [173] with a bulky mediastinal mass and occasionally with supraclavicular lymphadenopathy and shows an inferior survival as compared to cHL, PMBL or DLBCL.The diagnosis of MGZL is based both on morphological criteria (e.g., high number of tumor cells) and immunohistological findings (expression of >1 B-cell marker in a significant percentage of neoplastic cells) [174] (Fig. 5). Cases with otherwise typical nodular sclerosis cHL that express CD20 but are negative for other B-cell markers should not be designed as MGZL [3]. MGZL is rarely EBV-positive [175]. The features of MGZL as compared to cHL and PMBL are shown in Table 2. Cases with morphologic and immunophenotypic features similar to MGZL, but occurring outside the mediastinum, show different gene expression profiles and genetic alterations [169, 176]. Thus, they should be classified as DLBCL, NOS.

**Table 2:** Features of mediastinal gray zone lymphoma compared to cHL and primary mediastinal large B-cell lymphoma.

	Classical Hodgkin lymphoma (cHL)	Primary mediastinal large B-cell lymphoma (PMLBCL)	Mediastinal gray zone lymphoma (MGZL)
Clinical features	• Bimodal age distribution • Slight female predominance in nodular sclerosis • Frequent mediastinal involvement in nodular sclerosis • Most cases in stage I/II, in nodular sclerosis	• Young adults with female predominance (2:1) • Bulky mediastinal presentation (about 50%) • Rare involvement at unusual sites (kidneys, adrenals, CNS)	• Young adults with slight male predominance • Frequent mediastinal involvement with bulky disease • Primary extra-mediastinal presentation is better classified as DLBCL, NOS
Morphology	• Infrequent H-RS cells embedded in the typical cellular background (lymphocytes, eosinophils, histiocytes, plasma cells) • Collagen bands delimiting cellular nodules in nodular sclerosis • Syncytial variant of nodular sclerosis (rich in H-RS cells)	• Medium to large tumor cells with clear cytoplasm • Increase of reticular fibrosis leading to “compartmental” growth of tumor cells • Occasional H-RS like cells	• About 70% of cases shows a “cHL like morphology” resembling nodular sclerosis • About 30% mimic PMLBCL (monomorphic proliferation of medium-large neoplastic cells, pauci-cellular inflammatory infiltrate)
Immunophenotype	CD20− (rarely positive in a percentage or in most tumor cells), CD19−, CD79a−, CD79b−, PAX5+ (weak), CD30+ (strong), CD15+/−, MUM1/IRF4+, BCL6−, EBV −/+	CD20+, CD19+, CD79a+, CD79b+, PAX5+, BCL6+/−, CD30+ (weak/partial expression), CD23+ (>50%), MUM1/IRF4+/−, EBV−	Uniform and strong expression of >1 B-cell marker (CD20, CD19, CD79a, CD79b), PAX5*+* (strong), CD30+, MUM1/IRF4+, BCL6+, EBV− (rarely positive)
Genetics and molecular features	• JAK/STAT and NF-kB activation • Mutations in NF-kB inhibitors (*TNFAIP3*, *NFKBIE*, *NFKB1A*) • CN gains of *REL*, *JAK2* and *PD-L1/2* (9p24 amplification) • Mutations of *JAK1/3, STAT3/5B/6, SOCS1* • *CIITA* translocation	• JAK/STAT, NF-kB activation • CN gains of *REL, PD-L1/2* and *JAK2* (amplifications of 2p16 and 9p24) • Loss of *TNFAIP3, NFKBIE, EZH2, IL4R, GNA13* • *STAT6* mutations	• JAK/STAT and NF-kB activation • CN gains of *REL, PD-L1/2* and *JAK2* • Mutations in *SOCS1, TNFAIP3, NFKBIE, GNA13, XPO1* and *B2M* with loss of MHC-1 and MHC-2 expression • Lack of *BCL2* and *BCL6* translocations

*H-RS* Hodgkin and Reed-Sternberg cells.

### Multiple myeloma/plasma cell neoplasms

Non-IgM monoclonal gammopathy of undetermined significance (non-IgM MGUS) usually represents a precursor of MM [177]. Monoclonal gammopathy of renal or clinical significance (MGRS and MGCS) is characterized by a plasma cell or B-cell proliferation not meeting criteria for malignancy but secreting a monoclonal immunoglobulin or immunoglobulin fragment leading to kidney injury [178, 179]. The ICC describes MGRS/MGCS as a clinical feature of non-IgM MGUS whilst the WHO-HAEM5 regards it as an entity.

In the ICC classification [3], the term MM replaces that of “plasma cell myeloma” of WHO-HAEM4. The WHO-HAEM5 continues to use the term PCM. Based on progresses in cytogenetic/FISH studies, the ICC recognizes four mutually exclusive cytogenetic entities: (1) MM with *CCND* family translocations; (2) MM with *NSD2* translocation; (3) MM with *MAF* family translocation; and (4) MM with hyperdiploidy. MM without cytogenetic abnormalities is defined as a separate entity named MM, NOS (Table 1). Such a distinction will likely facilitate the evaluation of response to new drugs, including immunomodulatory agents, proteasome inhibitors, monoclonal antibodies and CAR-T cells, according to cytogenetic features. These drugs have significantly improved the survival of patients with MM [180]. Translating mutational studies into classification was felt premature because of the frequent subclonal evolution and spatial genomic heterogeneity typical of MM/PCM [181–184].

The t(11;14), the most common cytogenetic abnormality (20–30%) in MM/PCM, leads to hyperexpression of cyclin D1 and correlates with increased sensitivity to venetoclax [185]. Translocation t(4;14) (12–15% of patients) [186] is specific to MM/PCM and deregulates the *FGFR3* and *NSD2* genes, the latter being responsible for poor prognosis [186]. Concomitant 1p32 deletion can significantly worsen the prognostic impact of t(4;14) [187]. Interestingly, the widespread use of bortezomib may have contributed to the reduction of the unfavorable prognosis of t(4;14) [188]. The translocation t(14;16) involving *cMAF* occurs at a much lower frequency (3.5%) [186] and its prognostic impact yet remains controversial [186, 189, 190]. Hyperdiploidy accounts for about 55% of cases [186]. The trisomies preferentially affect chromosomes 3, 5, 7, 9, 11, 15, 19, and 21. However, only trisomies 3 and 5 are associated with a better prognosis whilst trisomy 21 has an unfavorable impact [191]. The 17p deletion associates with high-risk, especially when the clone size is 55–60% by FISH [192] and the patients harbor a “double-hit” biallelic inactivation of *TP53* [193].

Currently, MM/PCM patients receive similar treatment independently by the risk category. However, patients with high-risk disease still represent an unmet medical need with poor prognosis (death within the first 3 years from diagnosis) and should be identified for choosing the most efficient treatment strategy that maximizes the depth of response [194]. Inclusion of MM/PCM genetic categories in the classification may help to accelerate this process. The increasing importance of measurable residual disease [195] or PET/CT [196] in evaluating prognosis and risk stratification in MM/PCM is also recognized.

Smoldering/asymptomatic MM/PCM lacking features of active disease (SLiM CRAB criteria) [42] exhibits broad variability in progression to active MM/PCM. Patients suited for early therapeutic intervention are selected according to risk stratification models [197]. Solitary plasmacytomas of bone and primary extramedullary plasmacytomas have low-moderate risk for progression to MM/PCM [198]. Diagnosis, especially of solitary plasmacytomas of bone should be based on <10% clonal plasma cells detected by flow cytometry since this criterion is prognostically relevant [199].

Primary amyloidosis is termed immunoglobulin-related (AL) amyloidosis according to WHO-HAEM5 and Ig light chain (AL) amyloidosis according to ICC. The latter also emphasizes the importance of separating systemic AL amyloidosis from the localized form (Table 1), a rare disorder with excellent prognosis rarely progressing to systemic AL amyloidosis [200]. The WHO-HAEM4 recognized under the “plasma cell neoplasm with paraneoplastic syndrome” the POEMS and TEMPI syndromes as provisional entities. The TEMPI syndrome is characterized by telangiectasias, elevated erythropoietin, erythrocytosis, monoclonal gammopathy, perinephric fluid collection and intrapulmonary shunting [201]. Its diagnosis is mainly based upon clinical and imaging studies and it is now a defined entity in WHO-HAEM5. The AESOP syndrome is a newly recognized syndrome characterized by adenopathy with Castleman-like features and extensive skin patch overlying a plasmacytoma [202] that is regarded as an entity in WHO-HAEM5 but not in ICC.

## Conclusions

The clinico-pathological, immunophenotypic and molecular informations gained during the past 5 years in the field of lymphoid neoplasms have contributed to refining the diagnostic criteria of several entities, to upgrade entities previously defined as provisional and to identify new entities. This is reflected in the changes reported in the WHO-HAEM5 and ICC classifications as compared to WHO-HAEM4. However, in several areas (e.g., DLBCL, NOS), incorporation of the molecular data into the new classifications was still regarded as premature. Our comparative report of the WHO-HAEM5 and ICC classifications of mature B-cell neoplasms may hopefully serve as a tool to facilitate the work of pathologists, hematologists and researchers involved in the diagnosis and treatment of lymphomas.

However, two additional comments may be worthy. First, after more than 20 years, we have once again two classifications. The hope is to come as soon as possible to a unifying approach since there is urgent need of a common language, which can be shared by the international community. This is in the interest of patients, clinicians and pathologists. Secondly, both the WHO-HAEM5 and ICC are based increasingly on molecular data, which implies the need for a network of reference centers, where expertise and facilities are available. In fact, the achievement of the correct diagnosis, the costs for the techniques applied may not be affordable at the community hospital level. The risk is that such a specialized approach may penalize developing countries. The latter should be supported on both economic and technical grounds. One way of achieving this goal could be to devote efforts for education [203], technology transfer and the production of new monoclonal antibodies against mutated epitopes that may serve as surrogates for molecular studies, as those we now use to diagnose some entities, such as *NPM1*-mutated AML [204] or ALK+ anaplastic large cell lymphoma [205].
